# 

**Published:** 1864-02

**Authors:** 


### CIRCULAR.

SURGEON-GENERAL'S OFFICE,
RICHMOND, Oct. 31, 1863.

The following Prospectus meets with the hearty approval of the Surgeon-General, and the medical officers of the Confederate States service are earnestly requested to co-operate in the undertaking, and to forward their names, enclosing subscriptions, with as little delay as practicable.

S. P. MOORE, *Surgeon-General,*

### CONFEDERATE STATES
### MEDICAL AND SURGICAL JOURNAL,

**Published under the auspices of the Surgeon-General.**

**PRICE, $10 A YEAR IN ADVANCE.**

### PROSPECTUS.

It is proposed to commence the publication of a medical periodical in this city on the 1st of January next, and the co-operation of the army and navy medical staff, and of the profession at large, is respectfully solicited.

The responsible position which the Southern medical profession holds before the country demands that there should be some exponent of its unprecedented efforts, under the most adverse circumstances, during this war. Having free access to the reports and archives of the Medical Department, and acting in concert with the Association of Army and Navy Surgeons, this journal purposes to be the impartial representative of the profession, by collecting and elaborating the valuable results of its labors. Through this medium also we can be brought into communication with our brethren in other countries, and a chronicle of medical science, carefully collated from recent English and Continental periodicals, will be a leading feature. In short, no effort will be spared by its conductors to lay the foundation of a Southern Medical Literature on a firm and enduring basis.

The CONFEDERATE STATES MEDICAL AND SURGICAL JOURNAL will be published monthly, in quarto form, and issued regularly to subscribers, who have paid in advace, at TEN DOLLARS A YEAR.

Communications intended for insertion will be addressed to Editor of "Confederate States Medical and Surgical Journal," care of Ayres & Wade, Richmond.

All persons who favor the enterprise, whether in or out of the army, are requested to act as agents. Subscription and other letters upon business should be addressed to AYRES & WADE,
Publishers and Proprietors,
Richmond, Va.

